# On the controllability of a singular nonregular methaniser system

**DOI:** 10.1038/s41598-023-32344-7

**Published:** 2023-04-17

**Authors:** Zied Tmar, Taieb Wafi, Mongi Besbes

**Affiliations:** 1 University of Carthage, ISTIC, Tunis, 1054 Tunisia; 2 Société Tunisienne de l’Electricité et du Gaz, Tunis, 1003 Tunisia

**Keywords:** Energy science and technology, Engineering

## Abstract

The control and command of singular systems of non-regular type pose very complex problems for automation engineers. The classic concepts of controllability are not applicable because of the non-regularity of the response of such systems whose internal states are no longer controllable in the temporal or frequency plane. New concepts are rather considered such as R-Controllability and Imp-Controllability combined with $H_2/H_\infty$ type stabilization approaches. This paper proposes a new synthesis of a $H_2/H_\infty$ controller, written in terms of LMI-like matrix inequalities, dedicated to this type of system. The simulation results, of the controller synthesized on methanation plant are encouraging and have led to better performance compared to other scientific publications in the field.

## Introduction

Methane production systems have known in recent years a particular importance due to the growing need for the energy production without environmental consequences on the one hand, and also thanks to the search for ways of recycling waste and polluting materials for the inevitable sanitation located in all human presence places. A biodigester is a biologic system for recovering organic waste used to produce combustible gas (biogas) and fertilizer (digestate). The particularity of the biodigester is that the degradation is carried out by two types of bacteria in two successive phases in an oxygen-deprived environment, we speak of anaerobic fermentation^1^.

In order to optimize the operation of a biodigester, several researchers tried to find a faithful representative model to describe the actual operation of the process, we can cite here the works of Andrews et al.^2^, Bastin et al.^3^, Batstone et al.^4^, Bernard et al.^5^ and Hess^6^ as remarkable attempts. Despite the existence of proposed models, their exploitation was very difficult given their complexity^7–9^, which led other researchers to try to develop models that can be used for control process purposes^10^.

We notice, in the works which propose control means for the biodigester, an obvious negligence of a controllability study of the global system and its states (see for instance^3,11–13^). This poses a big question mark on the technical feasibility on the one hand and the usefulness of the results found on the other hand.

The reduced model used by Petre et al.^14^ (which will be adopted in this paper), despite its strong non-linearity, represents a new alternative for controlling a digester and following its evolution of its internal states which are difficult to control by conventional approaches^15–17^, let us quote^18–22^. In this context, in this paper, we will try to fill this gap by proposing a real-time control approach of the soft type which makes it possible to monitor the state to determine the state of controllability and the regularity of the bioreactor despite the strong non linearities and observed singularities. The determination of a command for a nonlinear noncontrollable system is possible under certain conditions, see the works of Marx^23^, Zerrougui^24^ and Draa et al.^25^. We propose a solution to this problem, based on the resolution of linearized matrix inequalities in the LMI form with control concepts $H_2/H_\infty$.

All cited works have preferred to linearize the system and study it around an operating point with the use of conventional control techniques. Some of the researchers have proposed the elimination of non-linearities by a Lipschitz function, this is likely to oversimplify the complexities inherent in the system. In the work of Petre et al.^14^, a linearization of the inputs/outputs is carried out despite the uncontrollability of two states of the system. In our work we preferred to rewrite the nonlinear equations of the system in the form of a singular system descriptor by a change of variable and thus take into account the problems of nonlinearity. The synthesis of the controller also has the advantage of considering a singular non-regular system with uncertainties bounded in norm, it is the first time that this type of problem is stated in this way to our knowledge. The control laws have made it possible to give the system better stability margins and very good control performance with a practically applicable control, which is not the case in other publications.

This paper is divided into three parts in addition to this introduction and a conclusion. In the second part, a detailed description of the evolution of the biological process of methane generation is presented. The role of each compartment of the installation is specified. The chemical equations which govern the process are written by specifying the chemical and biological components generated at each phase, the generation and the dispartition of acidogenic and methanogenic bacteria thus causing the loss of control of one of the states of the system. The second part ends with a setting in differential equations of type differential-algebraic equation (DAE) and an identification of the parameters of the system to be controlled. From the modeling, the third part is a study of the controllability of the singular system obtained. The concepts of R-Controllability and Imp-Controllability are introduced to apprehend new approaches to the synthesis of a stabilizing controller of a non-controllable system. In the fourth part, the synthesis of the controller is declined in terms of LMI written in an approach of $H_2/H_\infty$ stabilization for a singular non-regular system seat of an uncertainty bounded in norm. The paper ends with a conclusion.

## The methanation process and its modeling

### Process description

**Figure 1:**
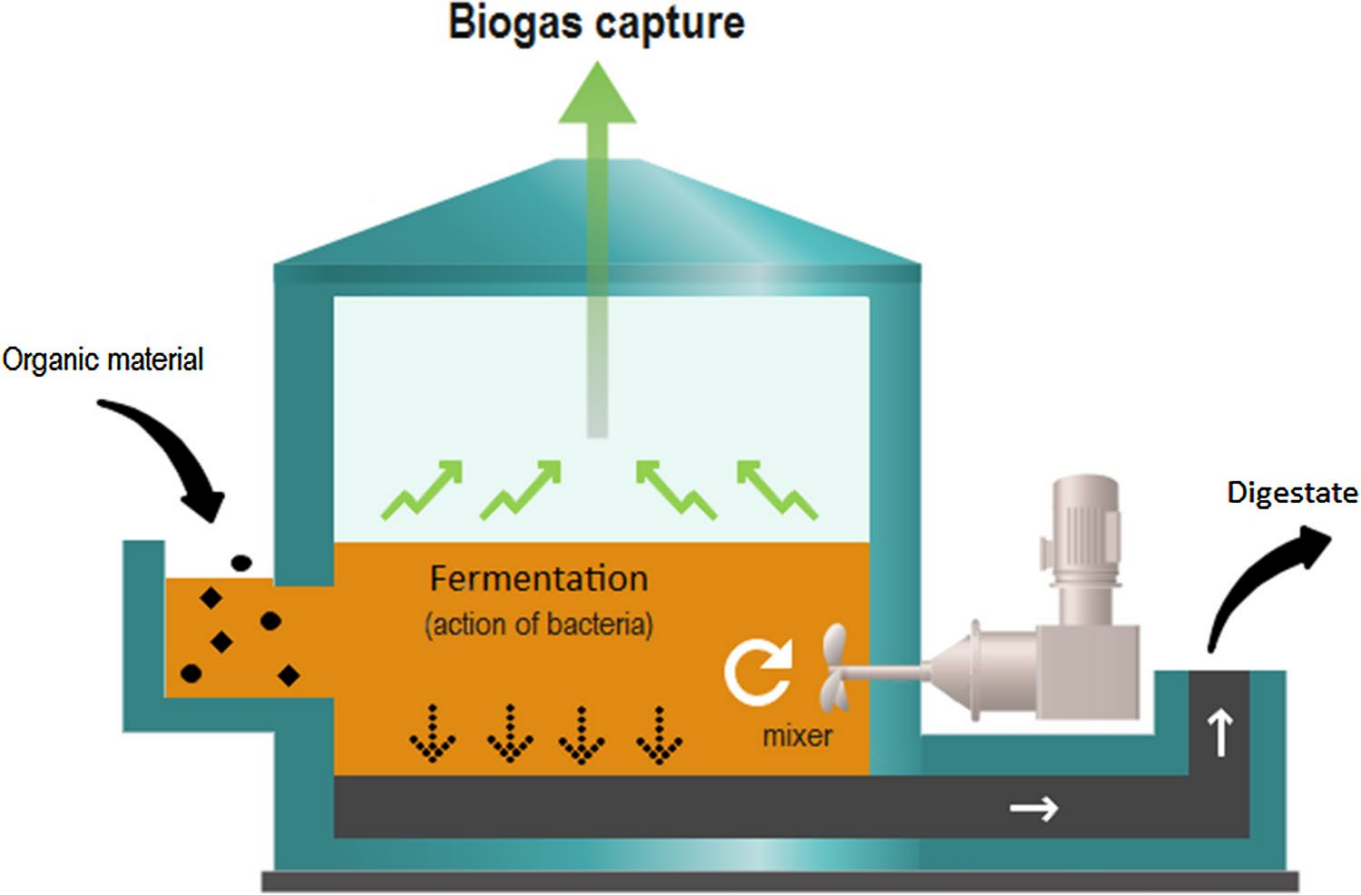
Methanizer (or anaerobic digestion) process, conditions and stages.

A methanizer (or anaerobic digestion), Fig. 1, is a natural process of recycling fermentable organic matter, in an oxygen-free environment, due to the action of micro-organisms which transform organic matter into biogas and digestate. This transformation is broken down into 4 major steps: hydrolysis, acidogenesis, acetogenesis and methanogenesis. It occurs naturally in certain environments, such as marshes, but can be implemented voluntarily by humans in dedicated units called “digesters” and under controlled conditions (a temperature maintained at $38^{\circ }$C, watertight, absence of oxygen, low pressure, etc.).

The biogas generated is composed of 60% methane ($CH_4$) and 40% carbon dioxide ($CO_2$), which can be recovered in the form of energy in electricity and heat thanks to a cogeneration engine, in biofuel or which can be injected into the natural gas network after a purification step, becoming biomethane or fuel biomethane (bioGNV). In energy terms, $1m^3$ of methane corresponds to 10kWh. The decomposition of organic matter also provides a product rich in humus and partially stabilized, called digestate, which can then be used as a natural and organic fertilizer for agriculture. In the case of anaerobic digestion, real chains of mineralization can be formed during which various groups of bacteria take turns to transform organic polymers to simpler molecules such as $CO_2$, $H_2$, $H_2O$, $CH_4$, etc. It is possible to distinguish three stages during this associative life without oxygen. Each step is carried out by a different type of bacteria. These bacteria are associated and complementary from a metabolic point of view, and form true symbiotic communities. Initially, polysaccharide complex organic materials such as cellulose, pectin, chitin, undergo fermentation. These fermentations release a wide variety of organic acids and alcohols, but also $CO_2$ and $H_2$. The derived organic metabolites can in turn be converted in a second step by the group of acetogenic bacteria. Acetic acid is the main product of this fermentation. Acetogenic bacteria occupy an intermediate niche in anaerobic populations between the fermentants which precede them and the methanogens, the third group of strict anaerobes which succeed them. Methanogenic bacteria, strictly anaerobic, lead to the production of methane from a mixture of carbon dioxide and hydrogen. These bacteria reduce $CO_2$ (or $HCO_3^-$) to methane. This pathway is energy-generating and coupled to ATP synthesis. This metabolism can therefore be considered as an example of autotrophy. The simplest scheme of methane production is as follows:

1 $$\begin{aligned} CO_2 + 4H_2 \longrightarrow CH_4 + 2H_2O \end{aligned}$$

#### Note 1

Bacteria disparition make the loss of controllability.

The energy and the reducing power come from a chemical reaction carried out from mineral substances. This is called chemolithotrophy.

#### Note 2

**Figure 2:**
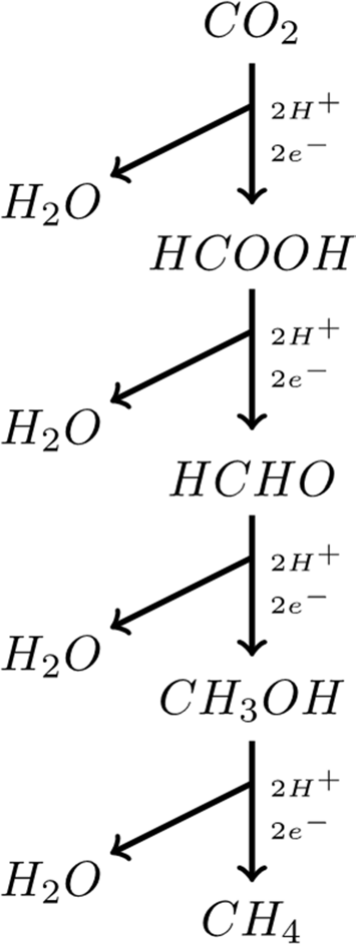
Methanogenesis by $CO_2$ reduction.

The transition from $CO_2$ to $CH_4$ (Fig. 2) takes place by four successive reductions but can also start from methanol or formic acid. The electrons can come from $H_2$ or even, more rarely, from iron ($F_eO$).

#### Note 3

The production of methane can also occur from the reduction of molecules other than carbon dioxide. Oxidation intermediates, such as methanol or formic acid, can be used. In the case of acetoclastic methanogenesis, the reduction takes place from acetic acid:

2 $$\begin{aligned} CH_3COOH \longrightarrow CH_4 + CO_2 \end{aligned}$$

**Figure 3:**
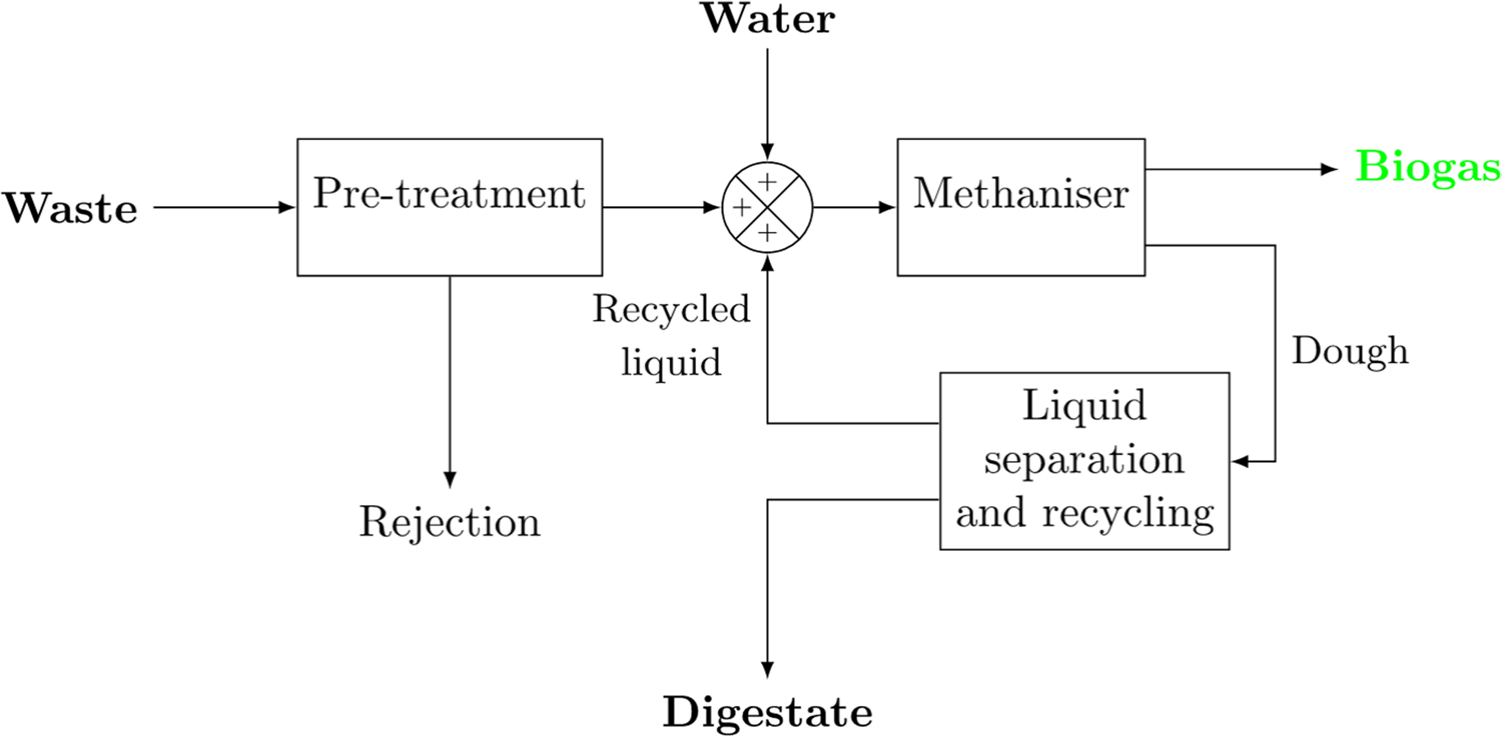
Block diagram of a biogas plant.

Both types of methanogenesis are related to bacteria from the Archaebacteria group. The formation of methane is linked to cooperative biological systems that continuously provide hydrogen and carbon dioxide or organic acids. Conversely, by removing hydrogen from the medium, methanogens thermodynamically favor the fermentations located upstream in the degradation chain. One of the particularities of these original metabolic pathways is the role played by cofactors which only exist in methanogens. These cofactors are sensitive to the presence of oxygen. Simple traces of oxygen in the medium therefore kill methanogenic bacteria. The doubling times are very different depending on the substrate used, from a few hours for hydrogenophilic bacteria to several days for acetoclast bacteria. The affinity constants with respect to the substrates are also very variable, thus very low levels of acetate tend to favor the presence of Methanothrix in anaerobic ecosystems. A perfect control of the hydraulics of the three-phase system is necessary to avoid non-fluidized zones, see Fig. 3.

Controlling bacterial mass is also necessary, so as to avoid too much lightening of the support material by the biomass which agglomerates there due either to an abnormal expansion of the bed, or to a clogging of this one. which would lead in both cases to losses of sludge and support materials.

**Figure 4:**
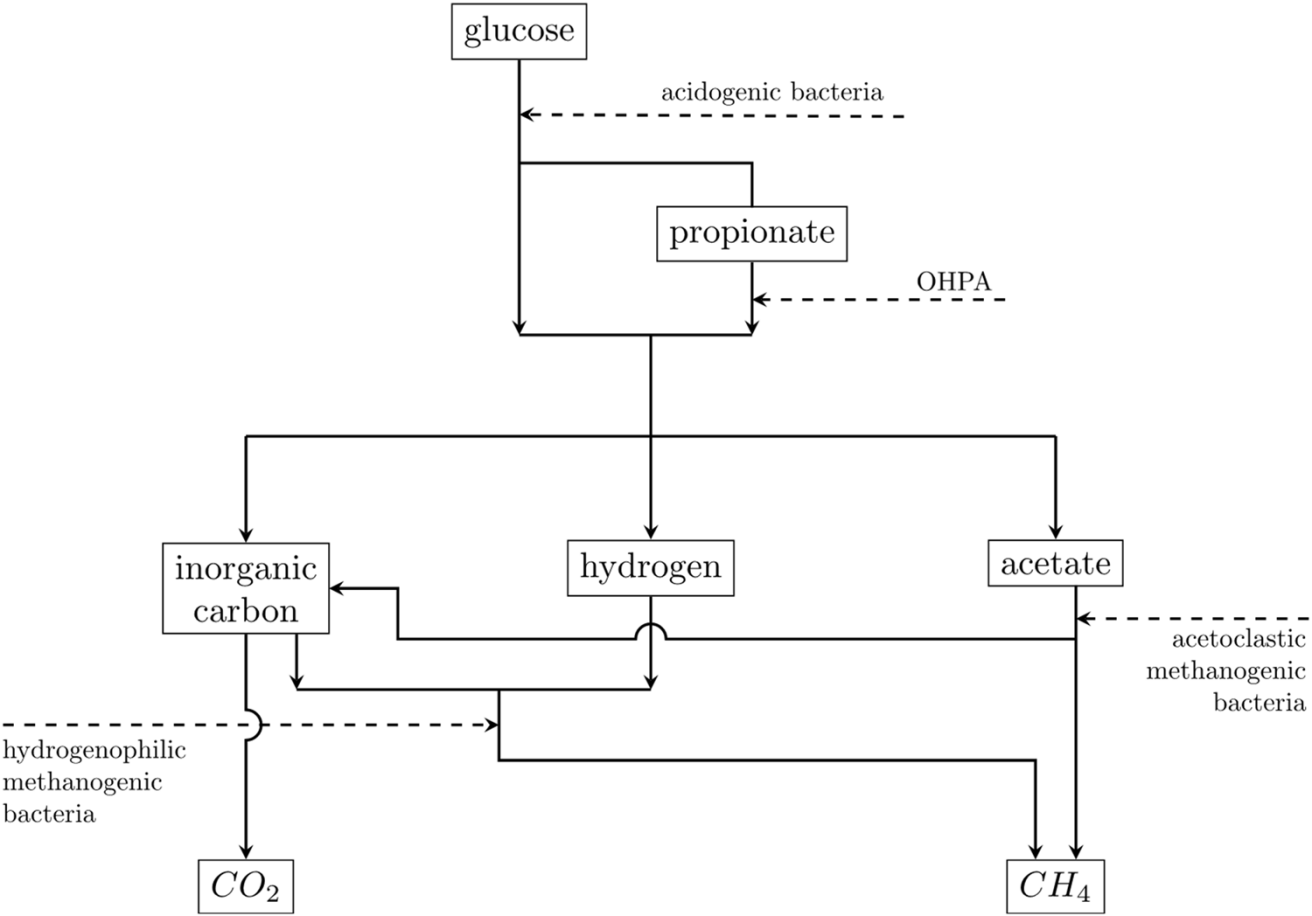
Scheme of the anaerobic digestion ^3^.

A methanizer can then be represented (see Fig. 4) by a multi-stage process whereby several functional groups of bacteria decompose the organic matter in the absence of oxygen through a series of chemical, physicochemical and biochemical reactions and produce carbon dioxide and usable methane ^5^.

Four metabolic paths can be identified: two for acidogenesis and two for methanisation^3–5^.

### Process model

The most known model of the described process is a generalized methanizer model ADM1 (Anaerobic Digestion Model No.1)^10^, described by differential equations contains 32 dynamic concentration state variables ^4^. Since the anaerobic digestion is a complex process, a reduced order model is used for control purpose. In this paper, the mathematical model used in^14^ is considered, given by the following mass balance equations :

3 $$\begin{aligned} \dot{X}_1&= \varphi _1-dX_1 \end{aligned}$$

4 $$\begin{aligned} \dot{S}_1&= -k_1\varphi _1-dS_1+dS_{1,in} \end{aligned}$$

5 $$\begin{aligned} \dot{X}_2&= \varphi _2-DX_2 \end{aligned}$$

6 $$\begin{aligned} \dot{S}_2&= k_3\varphi _1 - k_2\varphi _2 - dS_2 \end{aligned}$$

7 $$\begin{aligned} \dot{S}_3&= k_4\varphi _1 + k_5\varphi _2 - dS_3 - Q_{CO_2} \end{aligned}$$

8 $$\begin{aligned} \dot{S}_4&= k_6\varphi _2 + k_7\varphi _1 - dS_4 - Q_p \end{aligned}$$

where $X_1$, $X_2$ are the concentrations of acidogenic and acetoclastic methanogenic bacteria respectively, $S_1, S_2, S_3$ are glucose, acetate and carbon dioxide respectively, and $S_4$ is the methane concentration; $\varphi _1$ and $\varphi _2$ are the rates of the first acidogenic and methanisation reactions respectively defined by $\varphi _i=\mu _iX_i=\alpha _iS_iX_i$ (for $i=1,2$), where $\alpha _1=\frac{\mu _1^0}{K_{M_1}+S_1}$ and $\alpha _2=\frac{\mu _2^0}{K_{M_2}+S_2+\frac{S_2^2}{K_{I_2}}}$.

Note that $\mu _1^0=0.2~h^{-1}$ and $\mu _2^0=0.5~h^{-1}$ are maximums values of the growth rates $\mu _{10}(t)$ and $\mu _{20}(t)$ evolving with respect to the time according to

9 $$\begin{aligned} \mu _{10}(t)&=\mu _1^0(1+0.1\sin (\frac{\pi t}{10})) \end{aligned}$$

10 $$\begin{aligned} \mu _{20}(t)&=\mu _2^0(1-0.1\cos (\frac{\pi t}{20})) \end{aligned}$$

$K_{M_1}=0.75~g/l$ and $K_{M_2}=4~g/l$ are saturation constants, while $K_{I_2}=21~g/l$ is the inhibitory constant.

$Q_p=c_p\varphi _2$ (with $c_p=0.32$), represents the methane gaseous outflow rate and $Q_{CO_2}=c_{CO_2}S_3$ (with $c_{CO_2}=0.4$), represents the carbon dioxide gaseous outflow rate.

The input control *u* of the process is composed of : the influent substrate concentration $S_{1,in}$ and the dilution rate $d=F_{in,d}/V_d$, ($F_{in,d}$ is the pollutant influent flow rate and $V_d$ is the anaerobic digester volume) :

11 $$\begin{aligned} u=\begin{pmatrix} u_1\\ u_2\end{pmatrix}= \begin{pmatrix} d \\ S_{1,in} \end{pmatrix} \end{aligned}$$

Finally, $k_i (i = 0,...,7)$ are yield coefficients having the values^11^ : $k_1=3.2; k_2=16.7; k_3=1.035; k_4=1.194; k_5=1.5; k_6=3; k_7=0.113$. Using the rate expression, equations from (3) to (8) can be rewritten as following

12 $$\begin{aligned} \dot{X}_1&= \alpha _1S_1X_1-u_1X_1 \end{aligned}$$

13 $$\begin{aligned} \dot{S}_1&= -k_1\alpha _1S_1X_1-u_1S_1+u_1u_2 \end{aligned}$$

14 $$\begin{aligned} \dot{X}_2&= \alpha _2S_2X_2-u_1X_2 \end{aligned}$$

15 $$\begin{aligned} \dot{S}_2&= k_3\alpha _1S_1X_1 - k_2\alpha _2S_2X_2 - u_1S_2 \end{aligned}$$

16 $$\begin{aligned} \dot{S}_3&= k_4\alpha _1S_1X_1 + k_5\alpha _2S_2X_2 - u_1S_3 - c_{CO_2}S_3 \end{aligned}$$

17 $$\begin{aligned} \dot{S_4}&= k_6\alpha _2S_2X_2 + k_7\alpha _1S_1X_1 - u_1S_4 - c_p\alpha _2S_2X_2 \end{aligned}$$

These last equations stand for a state space representation of the bioprocess using six states, if we denote by $$x=\begin{bmatrix} X_1&S_1&X_2&S_2&S_3&S_4\end{bmatrix}^T$$ the state vector, we obtain the correspondent matrix state equation $\dot{x}=f(x,u)$. One can see that this system is a very complex nonlinear system for which we aren’t able to easily determine the controllability properties in order to design an appropriate control. For the purpose to study this controllability we give in the next sections a way to bypass the problem.

### Singular form of the process model

Let’s put the following combinations of the system states :

18 $$\begin{aligned} \zeta _1&=X_1 \end{aligned}$$

19 $$\begin{aligned} \zeta _2&=k_1X_1+S_1 \end{aligned}$$

20 $$\begin{aligned} \zeta _3&=k_3S_1+k_1k_2X_2+k_1S_2 \end{aligned}$$

21 $$\begin{aligned} \zeta _4&=-k_3X_1+k_2X_2+S_2 \end{aligned}$$

22 $$\begin{aligned} \zeta _5&=-k_4X_1-k_5X_2+S_3 \end{aligned}$$

23 $$\begin{aligned} \zeta _6&=-k_7X_1-(k_6-c_p)X_2+S_4 \end{aligned}$$

it means that the relation between $\zeta$ and *x* is given by a matrix *E* :

24 $$\begin{aligned} \zeta =\begin{bmatrix} \zeta _1\\ \zeta _2\\ \zeta _3\\ \zeta _4\\ \zeta _5\\ \zeta _6 \end{bmatrix}=\begin{bmatrix} 1 &{} 0 &{} 0 &{} 0 &{} 0 &{} 0\\ k_1 &{} 1 &{} 0 &{} 0 &{} 0 &{} 0 \\ 0 &{} k_3 &{} k_1k_2 &{} k_1 &{} 0 &{} 0\\ -k_3 &{} 0 &{} k_2 &{} 1 &{} 0 &{} 0 \\ -k_4 &{} 0 &{} -k_5 &{} 0 &{} 1 &{} 0 \\ -k_7 &{} 0 &{} -(k_6-c_p) &{} 0 &{} 0 &{} 1 \end{bmatrix}\begin{bmatrix} X_1 \\ S_1 \\ X_2 \\ S_2 \\ S_3 \\ S_4 \end{bmatrix}=Ex \end{aligned}$$

where we can notice that *E* is a singular matrix since $rank(E)=5<6$.

One can see that, on the one hand, the derivative of $\zeta$ with respect to the time is related to $\dot{x}$ using (24) as $\dot{\zeta }=E\dot{x}$ and, on the other hand, $\dot{\zeta }_i$ (components of the vector $\zeta$) are expressed using equations from (12) to (17) in the derivatives of equations from (18) to (23)

25 $$\begin{aligned} \dot{\zeta }_1&=\alpha _1S_1X_1-u_1X_1 \end{aligned}$$

26 $$\begin{aligned} \dot{\zeta }_2&=-k_1u_1X_1-u_1S_1+u_1u_2 \end{aligned}$$

27 $$\begin{aligned} \dot{\zeta }_3&=-k_3u_1S_1-k_1k_2u_1X_2- k_1u_1S_2+k_3u_1u_2 \end{aligned}$$

28 $$\begin{aligned} \dot{\zeta }_4&=k_3u_1X_1-k_2u_1X_2 - u_1S_2 \end{aligned}$$

29 $$\begin{aligned} \dot{\zeta }_5&=k_4u_1X_1+k_5u_1X_2 - u_1S_3 - c_{CO_2}S_3 \end{aligned}$$

30 $$\begin{aligned} \dot{\zeta }_6&=k_7u_1X_1+(k_6-c_p)u_1X_2 - u_1S_4 \end{aligned}$$

Our system (25) to (30), takes now the following form

31 $$\begin{aligned} \dot{\zeta }&=E\dot{x}=g(x,u) \end{aligned}$$

We can approximate the system (31) by linearization, to obtain a system having the following linear structure

32 $$\begin{aligned} E\dot{x}=Jx+Ku \end{aligned}$$

The exact linearization of the singular system (31) is based on the calculation of the jacobian matrices $\frac{\partial g_i}{\partial x_i}$ and $\frac{\partial g_i}{\partial u_i}$ which gives:

$$\begin{aligned} J=\begin{bmatrix} \alpha _1S_1-k_1u_1 &{} \beta _1X_1 &{} 0 &{} 0 &{} 0 &{} 0\\ -k_1u_1 &{} -u_1 &{} 0 &{} 0 &{} 0 &{} 0\\ 0 &{} -k_3u_1 &{} -k_1k_2u_1 &{} - k_1u_1 &{} 0 &{} 0\\ k_3u_1 &{} 0 &{} -k_2u_1 &{} - u_1 &{} 0 &{} 0\\ k_4u_1 &{} 0 &{} k_5u_1 &{} 0 &{} - u_1 - c_{CO_2} &{} 0\\ k_7u_1 &{} 0 &{} (k_6-c_p)u_1 &{} 0 &{} 0 &{} - u_1 \end{bmatrix} ; K=\begin{bmatrix} -X_1 &{} 0\\ -k_1X_1-S_1+u_2 &{} u_1\\ -k_3S_1-k_1k_2X_2 - k_1S_2+k_3u_2 &{} -k_1u_1\\ k_3X_1-k_2X_2 - S_2 &{} 0\\ k_4X_1+k_5X_2 - S_3 &{} 0\\ k_7X_1+(k_6-c_p)X_2 - S_4 &{} 0 \end{bmatrix} \end{aligned}$$

note that $\beta _1=K_{M_1}\alpha _1^2/\mu _1^0$. For the point $$x_0 = \begin{bmatrix}10&2&0.5&1&0&0\end{bmatrix}^T$$, we obtain

$$\begin{aligned} J=\begin{bmatrix} -0.097&{} 0.103&{} 0 &{} 0&{} 0&{} 0\\ -0.224 &{} -0.07 &{} 0 &{} 0 &{} 0 &{} 0\\ 0 &{} -0.073 &{} -3.741 &{}-0.224 &{} 0 &{} 0\\ 0.073 &{} 0 &{}-1.169 &{}-0.07 &{} 0 &{} 0\\ 0.084 &{} 0 &{}0.105 &{} 0 &{}-0.47 &{}0\\ 0.008 &{} 0 &{}0.188 &{} 0 &{} 0 &{}-0.07 \end{bmatrix}; ~~K=\begin{bmatrix} -2.9&{} 0\\ 24.42 &{} 0.07\\ 22.272 &{} -0.224\\ -0.939 &{} 0\\ 3.763 &{} 0\\ 0.864 &{} 0 \end{bmatrix} \end{aligned}$$

The obtained equation (32) represent a linear singular system. To study the controllability of this system we need to perform some preliminary stages, the first stage is a transformation into a canonical form called : the Kronecker-Weierstrass form, which is detailed in the beginning of the next section.

## Study of the process’s controllability

The study of the system controllability is very important when we need to control it. In the case of linear systems, this study was made easy thanks to the rank criterion, unfortunately in the case of nonlinear systems things are much more complicated, especially for highly complex nonlinear forms like that of the methanizer model. This controllability study, as we mentioned in the introduction, was neglected and ignored in almost all the works in literature dealing with methanizer control, and without explaining the reasons (see for example^3,7,11,13,14,20–22^). We give, in this paper, an approach which makes possible to study the controllability of the methanizer despite its singular form, this approach uses the results of Dai^26^, which in turn requires a canonical form of the singular system. In the following paragraph we explain how we can obtain this canonical form.

### Kronecker–Weierstrass form

The Kronecker-Weierstrass canonical form is described by

33 $$\begin{aligned} \begin{bmatrix}I&{}0\\ 0&{}N\end{bmatrix}Q^{-1}\dot{x}=\begin{bmatrix}A&{}0\\ 0&{}I\end{bmatrix}Q^{-1}x+\begin{bmatrix}B\\ D\end{bmatrix}u \end{aligned}$$

Transforming the system (32) into the form (33) comes down to find the unknwon matrices *N*, *A*, *B*, *D* and *Q*.

In order to compute the requested matrices we can, for example, follow the steps recommended by Gerdin^27^:

**Step 1 :** Compute the generalized Schur form^28^ of the matrix $\lambda E-J$ so that
  34 $$\begin{aligned} P_1(\lambda E-J)Q_1=\lambda \begin{bmatrix}E_1&{}E_2\\ 0&{}E_3\end{bmatrix}+\begin{bmatrix}J_1&{}J_2\\ 0&{}J_3\end{bmatrix} \end{aligned}$$
  In order to achieve this computation we can use the *QZ* factorization for generalized eigenvalues as indicated by Wilkinson^29^, the Matlab command is
  35 $$\begin{aligned}{}[EE,JJ,P_1,Q_1]&=qz[E,J] \end{aligned}$$
  where the results are
  $$\begin{aligned} EE&=\begin{bmatrix}E_1&{}E_2\\ 0&{}E_3\end{bmatrix}= \begin{bmatrix} -1 &{} 0 &{} -1.5 &{} 0 &{} -1.155&{} -0.303\\ 0 &{} -1 &{} -2.68 &{} 0 &{} -0.11 &{} -0.029\\ 0 &{} 0 &{} -55.989 &{} 3.353 &{} 0.048 &{} 1.034\\ 0 &{} 0 &{} 0 &{} -1.173e^{-16}&{} -1.0339 &{}0.048\\ 0 &{} 0 &{} 0 &{} 0 &{} -3.4859 &{} 0.078\\ 0 &{} 0 &{} 0 &{} 0 &{} 0 &{}-0.287 \end{bmatrix} \end{aligned}$$
  $$\begin{aligned} JJ&=\begin{bmatrix}J_1&{}J_2\\ 0&{}J_3\end{bmatrix}= \begin{bmatrix} 0.47&{} 0&{} 0.105&{} 0 &{} 0.081&{} 0.021\\ 0 &{} 0.07 &{} 0.188 &{} 0 &{} 0.008 &{} 0.002\\ 0 &{} 0 &{} 3.919 &{} -0.235 &{}-0.003 &{} -0.072\\ 0 &{} 0 &{} 0 &{} 1.08e^{-17} &{}0.072 &{}-0.003\\ 0 &{} 0 &{} 0 &{} 0 &{}0.244 &{}0.024\\ 0 &{} 0 &{} 0 &{} 0 &{} 0 &{}0.123 \end{bmatrix} \end{aligned}$$
  $$\begin{aligned} P_1=\begin{bmatrix} 0&{} 0&{} 0&{} 0&{} 1&{} 0\\ 0 &{} 0 &{} 0 &{} 0 &{} 0 &{} 1\\ 0 &{} 0 &{} -0.955 &{} -0.298 &{} 0 &{} 0\\ 0 &{} 0 &{}-0.298 &{} 0.955 &{} 0 &{} 0\\ -0.278 &{}-0.961 &{} 0 &{} 0 &{} 0 &{} 0\\ -0.961 &{}0.278 &{} 0 &{} 0 &{} 0 &{} 0 \end{bmatrix} ; Q_1=\begin{bmatrix} 0&{} 0 &{} 0&{} 0&{} 0.967&{} 0.254\\ 0 &{} 0 &{} 0 &{} 0 &{} 0.254 &{} -0.967\\ 0 &{} 0 &{} 1 &{} 0 &{} 0 &{} 0\\ 0 &{} 0 &{} 0 &{}-1 &{} 0 &{} 0\\ -1 &{}0 &{}0 &{}0 &{}0 &{} 0\\ 0 &{}-1 &{}0 &{}0 &{}0 &{}0 \end{bmatrix} \end{aligned}$$
  This means that
  $$\begin{aligned} E_1=\begin{bmatrix} -1 &{} 0 &{} -1.5 &{} 0 \\ 0 &{} -1 &{} -2.68 &{} 0 \\ 0 &{} 0 &{} -55.989 &{} 3.353 \\ 0 &{} 0 &{} 0 &{} -1.173e^{-16} \end{bmatrix} ; E_2=\begin{bmatrix} -1.155&{} -0.303\\ -0.11 &{} -0.029\\ 0.048 &{} 1.034\\ -1.0339 &{}0.048 \end{bmatrix} ; E_3=\begin{bmatrix} -3.4859 &{} 0.078\\ 0 &{}-0.287 \end{bmatrix} \end{aligned}$$
  and
  $$\begin{aligned} J_1=\begin{bmatrix} 0.47&{} 0&{} 0.105&{} 0 \\ 0 &{} 0.07 &{} 0.188 &{} 0 \\ 0 &{} 0 &{} 3.919 &{} -0.235 \\ 0 &{} 0 &{} 0 &{} 1.08e^{-17} \end{bmatrix} ; J_2=\begin{bmatrix} 0.081&{} 0.021\\ 0.008 &{} 0.002\\ -0.003 &{} -0.072\\ 0.072 &{}-0.003 \end{bmatrix} ; J_3=\begin{bmatrix} 0.244 &{}0.024\\ 0 &{}0.123 \end{bmatrix} \end{aligned}$$
**Step 2 :** Solve the generalized Sylvester equations (36) and (37) to get matrices *R* and *T*.
  36 $$\begin{aligned} E_1R+TE_3&=-E_2\end{aligned}$$
  37 $$\begin{aligned} J_1R+TJ_3&=-J_2 \end{aligned}$$
  The generalized Sylvester equations (36) and (37) can be solved, as indicated by Kagstrom^30^, from a linear system of equations having the form $WY=Z$ as follows
  38 $$\begin{aligned} \begin{bmatrix} I_p\bigotimes E_1 &{} E_3^T\bigotimes I_q\\ I_p\bigotimes J_1 &{} J_3^T\bigotimes I_q \end{bmatrix} \begin{bmatrix}vec(R)\\ vec(L) \end{bmatrix}=\begin{bmatrix}-vec(E_2)\\ -vec(J_2) \end{bmatrix} \end{aligned}$$
  under Matlab using the command $Y=W\backslash Z$. The results are
  39 $$\begin{aligned} R=\begin{bmatrix} 5.551e^{-17}&{} 3.19\\ -3.224 &{} 0.091\\ 0.558 &{} -0.017\\ 4.152 &{}-0.349 \end{bmatrix} \end{aligned}$$
  40 $$\begin{aligned} T=\begin{bmatrix} -0.571&{} -12.244\\ 0.464 &{} -0.134\\ -4.954 &{} 1.431\\ -0.297 &{} 0.086 \end{bmatrix} \end{aligned}$$
**Step 3 :** Get the form (33) according to
  41 $$\begin{aligned} P=\begin{bmatrix}E_1^{-1}&{}0\\ 0&{}J_3^{-1}\end{bmatrix}\begin{bmatrix}I&{}T\\ 0&{}I\end{bmatrix}P_1=\begin{bmatrix} -11.922&{} 0.236e^{+15}&{} -0.228e^{+15}&{} 0.731e^{+15}&{} -1&{} 0\\ -0.031 &{} 0.422e^{+15} &{} -0.408e^{+15} &{} 1.306e^{+15} &{} 0 &{} -1\\ 0 &{} -0.158e^{+15} &{} 0.152e^{+15} &{} -0.487e^{+15} &{} 0 &{} 0\\ 0.125 &{}-2.632e^{+15} &{}2.543e^{+15} &{} -8.139e^{+15} &{}0 &{}0\\ -0.361 &{}-4.161 &{}0 &{} 0 &{} 0 &{} &{}0\\ -7.818 &{} 2.258 &{}0 &{} 0 &{} 0 &{} &{}0 \end{bmatrix} \end{aligned}$$
  42 $$\begin{aligned} Q=Q_1\begin{bmatrix}I&{}R\\ 0&{}I\end{bmatrix}=\begin{bmatrix} 0&{} 0&{} 0&{} 0&{} 0.967&{} 0.254\\ 0 &{} 0 &{} 0 &{} 0 &{} 0.254 &{} -0.967\\ 0 &{} 0 &{} 1 &{} 0 &{} 0.558 &{} -0.017\\ 0 &{} 0 &{} 0 &{} -1 &{}-4.152 &{} 0.349\\ -1 &{} 0 &{} 0 &{} 0 &{}-5.551e^{-17}&{} -3.19\\ 0 &{} -1 &{} 0 &{} 0 &{}3.224 &{}-0.091 \end{bmatrix} \end{aligned}$$
  43 $$\begin{aligned} N=J_3^{-1}E_3= \begin{bmatrix} -14.286 &{} 0.552\\ 0 &{} -2.334 \end{bmatrix} ~~and~~A=E_1^{-1}J_1= \begin{bmatrix} -0.47 &{} 0 &{} 2.149e^{-18} &{} 0.0020\\ 0 &{} -0.07 &{} 2.776e^{-17} &{} 0.0036\\ 0 &{} 0 &{} -0.07 &{}-0.001\\ 0 &{} 0 &{} 0 &{} -0.092 \end{bmatrix} \end{aligned}$$
  44 $$\begin{aligned} \begin{bmatrix}B\\ D\end{bmatrix}=PK=\begin{bmatrix} 108&{} 0.677e^{+14}\\ 24 &{} 1.21e^{+14}\\ -5 &{}-0.452e^{+14}\\ -64 &{}-7.54e^{+14}\\ -100.5707&{} -0.2913\\ 77.8054 &{} 0.1580 \end{bmatrix} \Rightarrow B=\begin{bmatrix} 108&{} 0.677e^{+14}\\ 24 &{} 1.21e^{+14}\\ -5 &{}-0.452e^{+14}\\ -64 &{}-7.54e^{+14} \end{bmatrix} ; D=\begin{bmatrix} -100.5707&{} -0.2913\\ 77.8054 &{} 0.1580 \end{bmatrix} \end{aligned}$$

This makes the end of the unknown matrices determination stage in order to write the singular system in a canonical form. In what follows we exploit this canonical form to study the controllability of the system, this study is based on two types of controllability, one called R-controllability which corresponds to the slow subsystem and the other called Imp-controllability which corresponds to the fast subsystem.

### R-controllability

In the previous paragraph we managed to write the system (32) in the equivalent Kronecker-Weierstrass form (33), this last one can be splitted into two subsystems as written in the following:

45 $$\begin{aligned} \dot{\bar{x}}_1&=A\bar{x}_1+Bu \end{aligned}$$

46 $$\begin{aligned} N\dot{\bar{x}}_2&=\bar{x}_2+Du \end{aligned}$$

Where $\bar{x}_1\in \mathbb {R}^{n_1}$, $\bar{x}_2\in \mathbb {R}^{n_2}$, $A\in \mathbb {R}^{n_1\times n_1}$, $N\in \mathbb {R}^{n_2\times n_2}$ and $n_1 +n_2=n$. As defined by Dai^26^, the singular system (32) is R-controllable if the slow subsystem (45) is controllable, in other words if the following rank condition is satisfied

47 $$\begin{aligned} rank\begin{bmatrix}B&AB&\dots&A^{n_1-1}B\end{bmatrix}=n_1 \end{aligned}$$

in our case, $n_1=4$, we have $$rank\begin{bmatrix}B&AB&A^2B&A^3B\end{bmatrix}=3<4$$, we deduce that the slow subsystem (45) is not controllable, so the system (32) is not R-controllable.

### Imp-controllability

As defined also by Dai^26^, the singular system (32) is Imp-controllable if the fast subsystem (46) is Imp-controllable, in other words if the following rank condition is satisfied

48 $$\begin{aligned} rank\begin{bmatrix}E &{} 0 &{} 0\\ J &{} E &{} K\end{bmatrix}=n + rank[E] \end{aligned}$$

in our case, $n=6$ and $rank[E]=5$, we obtain $$rank\begin{bmatrix}E &{} 0 &{} 0\\ J &{} E &{} K\end{bmatrix}=11$$. Therefore, the system (32) is Imp-controllable.

### Controllability

Dai^26^ demonstrated that the singular system (32) is controllable if both slow and fast subsystems are controllable, in other words : if the system (32) is in the same time R-controllable and Imp-controllable. Because the R-controllability is not satisfied, although the Imp-controllability is there, the studied system (32) is not controllable. The approximate controllability^31–34^ was considered in some publications to come over the problem of controllability loss during the process evolution.

### Admissibility

For singular systems, the study of admissibility is more judicious than the study of the stability. However, in the literature, only stability was taken into account. Let’s remember that the stability notion known in the standard systems case is not sufficient. Therefore, we try here to verify regularity and impulse properties for the autonomous singular system correspondent to (32):

49 $$\begin{aligned} E\dot{x}=Jx \end{aligned}$$

The admissibility study of the system (49) uses the matrix $F=sE-J$ for which it’s clear that its determinant $det(sE-J)=0~\forall s$. We deduce that the system (49) is not regular. In the next section we carry out the design of a methanizer control despite the handicaps represented by its non-linearity, singularity, non-regularity.

## Process control design

In the previous section we proved that the system is not controllable, this disappointing result does not necessarily imply the impossibility of controlling the system because it concerns the total controllability. The controllability of our system is then only partial, just two states are becoming non-controllable after few hours of reaction because of the death of acidogenic bacteria in stage 1 and the death of methanogenic bacteria in stage 2, it’s in this spirit that we therefore try to find a control for the system.

In this section we present a stabilizing control synthesis method for the considered methaniser which is a nonlinear singular system. The existing non-linearity in the methanizer model is continuous and differentiable^35^, this non-linearity is transformed by the Hadamard lemma in a form well adapted to the synthesis of a control law by using of stability in the Lyapunov sense.

### Preliminaries

Consider the following singular system where in its first equation (50) we have different terms corresponding to a command, a non-linearity and disturbances

50 $$\begin{aligned} E\dot{x}(t)&=A_{\Delta }(x)x(t)+B_0u(t)+D_0f(t,F_Lx,u) + D_1w(t) \end{aligned}$$

51 $$\begin{aligned} y&=Cx(t)+ D_2w(t) \end{aligned}$$

where the initial state $x(0)=x_0$, $x(t)\in \mathbb {R}^n$ is the state vector, $u(t)\in \mathbb {R}^m$ is the control, $w\in \mathbb {R}^{n_w}$ is an exogenous disturbance vector, $y(t)\in \mathbb {R}^p$ is the output and $f(t,F_Lx,u)$ the non-linearity. Matrices $A_{\Delta }\in \mathbb {R}^{n\times n}$, $B_0\in \mathbb {R}^{n\times m}$, $C\in \mathbb {R}^{p\times n}$, $D_0\in \mathbb {R}^{n\times n_f}$, $D_1\in \mathbb {R}^{n\times n_w}$, $D_2\in \mathbb {R}^{p\times n_w}$, since the measuring instruments have good precision, the matrix $D_2$ is assumed to be zero, and $F_L\in \mathbb {R}^{n_f\times n}$.

By definition

52 $$\begin{aligned} A_{\Delta }(x)=A_0+ A_{\delta }(x) \end{aligned}$$

where $A_{\delta }$ is an indigenous disturbance. To simplify, we use the notation $A_{\Delta }$ for $A_{\Delta }(x)$ and $A_{\delta }$ for $A_{\delta }(x)$.

We assume the following hypothesis to be valid:

#### Hypothesis 1

The matrix $A_{\delta }$ is expressed as follows

53 $$\begin{aligned} A_{\delta }=D_{\Delta }F_{\Delta }E_{\Delta } \end{aligned}$$

#### Hypothesis 2

$A_{\Delta }$ is a Routh matrix, which mean that all its eigenvalues ($\lambda _i$) satisfy the condition

54 $$\begin{aligned} \mathscr {R}(\lambda _i)<0 \end{aligned}$$

#### Hypothesis 3

The matrix $F_{\Delta }$ is supposed to have bounded norm

55 $$\begin{aligned} F_{\Delta }^TF_{\Delta }<\gamma _{\Delta }^2I \end{aligned}$$

#### Hypothesis 4

The non-linearity *f*(*t*, *s*, *u*) is class $C^1$, $f(t,0,u)=0$ and verify :

56 $$\begin{aligned} \Vert \frac{\partial f(t,s,u)}{\partial s}\Vert \le K, ~K\in \mathbb {R}^+ \end{aligned}$$

In this case we can notice that

#### Remark 1

According to Khalil^36^ in lemma 2.2, if *f* is class $C^1$ satisfying (56) then *f*(*t*, *s*, *u*) is local Lipschitz.

#### Remark 2

Hypothesis 1 is stated because we suppose uncertainties inherent to the system are bounded in norm^37^. It’s not possible to consider eigenvalues of $A_{\Delta }$ with a positive real part which will make the system diverge so we need Hypothesis 2. Hypothesis 3 was put to ensure the robustness of the system.

From the Hypothesis 4 there exist two matrices $M_f\in \mathbb {R}^{n_f\times n_f}$ and $N_f\in \mathbb {R}^{m_f\times n_f}$ such that :

57 $$\begin{aligned} \frac{\partial f(t,s,u)}{\partial s}=M_fF(t,s,u)N_f ~~with~~ F^T(t,s,u)F(t,s,u)\le I \end{aligned}$$

According to the Hadamard lemma^38^, if *f*(*t*, *s*, *u*) verify (56), we obtain

58 $$\begin{aligned} f(t,F_Lx,u)=\int _0^1\frac{\partial f(t,s,u)}{\partial s}|_{s=\lambda F_Lx}F_Lxd\lambda =\int _0^1M_fF(s)N_fF_Lx(t)d\lambda \end{aligned}$$

### Synthesis of a stabilizing control law

Let’s consider the gain matrix $L\in \mathbb {R}^{n\times m}$ of the state feedback such that the singular closed-loop system, under the loop $u(t)=-Lx(t)$, is admissible for $w(t)=0$ and satisfy the performance $H_{\infty }$ ($J_{yw}<\gamma _n$) for a $\gamma _n>0$, when $w(t)\ne 0$.

By substituting the control $u(t)=-Lx(t)$, the sytem (50) becomes :

59 $$\begin{aligned} E\dot{x}(t)=A(x)x(t)+ D_1w(t) \end{aligned}$$

where

60 $$\begin{aligned} A(x)=(A_{\Delta }-LB_0)+D_0\int _0^1M_fF(s)N_fF_Lx(t)d\lambda \end{aligned}$$

#### Lemma 1

Let’s *U* and *V* two vectors with appropriate dimensions, then, for all real $\mu >0$ the following inequality is satisfied:

61 $$\begin{aligned} U^TV+V^TU\le \mu U^TU+\mu ^{-1}V^TV \end{aligned}$$

The next theorem gives sufficient stability conditions for the system (50)-(51) for $w(t)=0$ and $\Vert x(t)\Vert _2<\gamma _n\Vert w(t)\Vert _2$, for $w(t)\ne 0$.

**Theorem 1 **The system (59) is asymptotically stable for $w(t)=0$ and $\Vert x(t)\Vert _2<\gamma _n\Vert w(t)\Vert _2$, for $w(t)\ne 0$ if there exist a definite positive matrix $\bar{X}$, matrices *X*, *Q* and positive scalars $\mu$ and $\mu _{\delta }$ such that the following LMI is satisfied

62 $$\begin{aligned} \begin{bmatrix} \Sigma _{1}&{} D_1 &{} A_{\delta } &{} (E\bar{X}+QE^{T\bot }) &{} (E\bar{X}+QE^{T\bot })F_L^TN_f^T\\ *&{} -\gamma _n^2I &{} 0 &{} 0 &{} 0 \\ *&{} 0 &{} -\mu _{\delta }I &{} 0 &{} 0 \\ *&{} 0 &{} 0 &{} -(1+\mu _{\delta })^{-1}I &{} 0\\ *&{} 0 &{} 0 &{} 0 &{} -\mu I \end{bmatrix}<0 \end{aligned}$$

where

63 $$\begin{aligned} \Sigma _{1}=E\bar{X}A_0^T+QE^{T\bot }A_0^T+A_0\bar{X}E^T +A_0E^{T\bot T}Q^T- XB_0^T-B_0X^T+\mu D_0M_fM_f^TD_0^T \end{aligned}$$

In this case, the gain matrix *L* is given by $L=X^T(E\bar{X}+QE^{T\bot })^{-T}$.

#### Proof

Let’s consider the Lyapunov function: $V(x)=x^TE^TYx=x^TY^TEx$ where *Y* is a regular matrix verifying:

64 $$\begin{aligned} E^TY=Y^TE\ge 0 \end{aligned}$$

The derivative of *V*(*x*) along the solution of (59) is $\dot{V}(x)=(E\dot{x})^TYx +x^TY^TE\dot{x}$. Using the model (59) we obtain

$$\begin{aligned} \dot{V}(x)=[A(x)x+ D_1w]^TYx +x^TY^T[A(x)x+ D_1w] \end{aligned}$$

so we get

65 $$\begin{aligned} \dot{V}(x)=x^T[A(x)^TY+Y^TA(x)]x+w^TD_1^TYx+x^TY^TD_1w \end{aligned}$$

let’s put $$\xi =\begin{bmatrix} x\\ w\end{bmatrix}$$. Thus, the equation (65) becomes

$$\begin{aligned} \dot{V}(x)= \xi ^T\begin{bmatrix} A(x)^TY+Y^TA(x)+I &{} Y^TD_1\\ D_1^TY &{} -\gamma _n^2 \end{bmatrix}\xi -x^Tx+\gamma _n^2w^Tw \end{aligned}$$

If we have

66 $$\begin{aligned} \begin{bmatrix} A(x)^TY+Y^TA(x)+I &{} Y^TD_1\\ D_1^TY &{} -\gamma _n^2 \end{bmatrix}<0 \end{aligned}$$

then we obtain $\dot{V}(x)<\gamma _n^2w^Tw-x^Tx$, by integrating both sides of this inequality, we get

$$\begin{aligned} \int _0^\infty \dot{V}(\tau )d\tau <\int _0^\infty \gamma _n^2w^Twd\tau -\int _0^\infty x^Tx d\tau \end{aligned}$$

which is equivalent to $V(\infty )-V(0)<\gamma _n^2\Vert w\Vert _2-\Vert x\Vert _2$. Under the zero initial condition, we get $V(\infty )<\gamma _n^2\Vert w\Vert _2-\Vert x\Vert _2$ which is equivalent to $\Vert x\Vert _2<\gamma _n^2\Vert w\Vert _2$. If we put $W=Y^{-T}$, so $W^T=Y^{-1}$, pre-multiplying the first member of (66) by $$\begin{bmatrix} W &{} 0\\ 0 &{} I\end{bmatrix}$$, and post-multiplying it by $$\begin{bmatrix} W^T &{} 0\\ 0 &{} I\end{bmatrix}$$, gives

67 $$\begin{aligned} \begin{bmatrix} WA(x)^T+A(x)W^T+WW^T &{} D_1\\ D_1^T &{} -\gamma _n^2I \end{bmatrix}<0 \end{aligned}$$

The condition (64) is equivalent to $WE^T=EW^T\ge 0$. Using (52) and replacing *A*(*x*) with its expression given by (60) in  (67) we get

68 $$\begin{aligned} \begin{bmatrix} \boxed {\begin{matrix} WA_0^T+A_0W^T+WA_{\delta }^T+A_{\delta }W^T-WL^TB_0^T-B_0LW^T\\ +WW^T +W\int _0^1F_L^TN_f^TF(\lambda F_Lx,t)M_f^TD_0^Td\lambda \\ +D_0\int _0^1M_fF(\lambda F_Lx,t)N_fF_Ld\lambda W^T \end{matrix} } &{} D_1\\ &{} \\ D_1^T &{} -\gamma _n^2I \end{bmatrix}<0 \end{aligned}$$

If we partition the matrix in (68) as follows $$\begin{bmatrix} \Sigma _A &{} \Sigma _B\\ \Sigma _C &{} \Sigma _D \end{bmatrix}$$, applying Schur’s complement^28^, the condition (68) becomes $\Sigma _A-\Sigma _B\Sigma _D^{-1}\Sigma _C<0$, which means

69 $$\begin{aligned} WA_0^T+A_0W^T+WA_{\delta }^T+A_{\delta }W^T-WL^TB_0^T-B_0LW^T+W\int _0^1F_L^TN_f^TF(\lambda F_Lx,t)M_f^TD_0^Td\lambda \nonumber \\ +WW^T +D_0\int _0^1M_fF(\lambda F_Lx,t)N_fF_Ld\lambda W^T+\gamma _n^{-2}D_1D_1^T<0 \end{aligned}$$

If we put $U=M_f^TD_0^T$ and $V=\int _0^1F(\lambda F_Lx,t)N_fF_Ld\lambda W^T$ in Lemma 1, using (57) and (61) lead to

70 $$\begin{aligned} W\int _0^1F_L^TN_f^TF(\lambda F_Lx,t)M_f^TD_0^Td\lambda +D_0\int _0^1M_fF(\lambda F_Lx,t)N_fF_Ld\lambda W^T <\mu D_0M_fM_f^TD_0^T+\mu ^{-1}WF_L^TN_f^TN_fF_LW^T \end{aligned}$$

Let’s put the following condition:

71 $$\begin{aligned} WA_{\delta }^T+A_{\delta }W^T<0 \end{aligned}$$

Another time, if we put $U=W^T$ and $V=A_{\delta }^T$ in Lemma 1, using (57), (71) verifies

72 $$\begin{aligned} WA_{\delta }^T+A_{\delta }W^T<\mu _{\delta }WW^T+\mu _{\delta }^{-1}A_{\delta }A_{\delta }^T \end{aligned}$$

From (69), (70) and (72) we get

73 $$\begin{aligned} &WA_0^T+A_0W^T+WA_{\delta }^T+A_{\delta }W^T-WL^TB_0^T-B_0LW^T +W\int _0^1F_L^TN_f^TF(\lambda F_Lx,t)M_f^TD_0^Td\lambda \nonumber \\ &\quad +D_0\int _0^1M_fF(\lambda F_Lx,t)N_fF_Ld\lambda W^T +WW^T+\gamma ^{-2}_nD_1D_1^T <WA_0^T+A_0W^T-WL^TB_0^T-B_0LW^T \nonumber \\ &\quad +\mu _{\delta }WW^T+\mu _{\delta }^{-1}A_{\delta }A_{\delta }^T +\mu D_0M_fM_f^TD_0^T+\mu ^{-1}WF_L^TN_f^TN_fF_LW^T +WW^T+\gamma ^{-2}_nD_1D_1^T \end{aligned}$$

Now, if

74 $$\begin{aligned} WA_0^T+A_0W^T-WL^TB_0^T-B_0LW^T+\mu D_0M_fM_f^TD_0^T \nonumber \\ +\mu ^{-1}WF_L^TN_f^TN_fF_LW^T +\gamma ^{-2}_nD_1D_1^T+\mu _{\delta }^{-1}A_{\delta }A_{\delta }^T+(1+\mu _{\delta })WW^T<0 \end{aligned}$$

is verified, so (69) is verified. By applying the Schur’s complement ^28^ and introducing $X=WL^T$, (74) becomes

75 $$\begin{aligned} \begin{bmatrix} \boxed {\begin{matrix}WA_0^T+A_0W^T-XB_0^T-B_0X^T\\ +\mu D_0M_fM_f^TD_0^T\\ +\mu ^{-1}WF_L^TN_f^TN_fF_LW^T \end{matrix} }&{} D_1 &{} A_{\delta } &{} W\\ &{} &{} &{} \\ D_1^T &{} -\gamma ^2_nI &{} 0 &{} 0\\ A_{\delta }^T &{} 0 &{} -\mu _{\delta }I &{}0 \\ W^T &{} 0 &{} 0 &{} -(1+\mu _{\delta })^{-1}I\end{bmatrix}<0 \end{aligned}$$

By setting

76 $$\begin{aligned} W=E\bar{X}+QE^{T\bot } \end{aligned}$$

the inequality $WE^T=EW^T\ge 0$ is always true and by replacing (76) with (75) we get

$$\begin{aligned} \begin{bmatrix} \Sigma _{1}+\Sigma _2 &{} D_1 &{} A_{\delta } &{} E\bar{X}+QE^{T\bot }\\ D_1^T &{} -\gamma ^2_nI &{} 0 &{} 0\\ A_{\delta }^T &{} 0 &{} -\mu _{\delta }I &{} 0\\ (E\bar{X}+QE^{T\bot })^T &{} 0 &{} 0 &{} -(1+\mu _{\delta })^{-1}I\end{bmatrix}<0 \end{aligned}$$

with $$\Sigma _2 = \mu ^{-1}(E\bar{X}+Q
E^{T\bot })F_L^TN_f^TN_fF_L(E\bar{X}+QE^{T\bot })^T$$.

Applying Schur’s complement^28^ to this inequality we get (62). This proves the theorem.

### Simulation results

The numerical values for the system matrices are as following :

$$\begin{aligned} A_0=\begin{bmatrix} 0.0754545 &{} 0.1283471 &{} -0.07 &{} -0.07 &{} -0.07 &{} -0.07\\ -0.5354545 &{} -0.7047107 &{} -0.07 &{} -0.07 &{} -0.07 &{} -0.07\\ -0.07 &{} -0.07 &{} 0.0191509 &{} -0.0350966 &{} -0.07 &{} -0.07\\ 0.0805455 &{} 0.1352893 &{} -1.5588208 &{} -0.6528874 &{} -0.07 &{} -0.07\\ 0.1036727 &{} 0.1668264 &{} 0.0637264 &{} -0.0176448 &{} -0.47 &{} -0.07\\ -0.0535636 &{} -0.0475868 &{} 0.1689245 &{} 0.0235412 &{} -0.07 &{} -0.07 \end{bmatrix} ; ~~A_{\delta }=\pm 10^{-2}A_0 \end{aligned}$$

$$\begin{aligned} B_0=\begin{bmatrix} -10 &{} 0\\ 33 &{} 0.07\\ -0.5 &{} 0\\ -1 &{} 0\\ 0 &{} 0\\ 0 &{} 0 \end{bmatrix} ; ~~D_0=\begin{bmatrix} 1 \\ 0 \\ 0 \\ 0 \\ 1 \\ 1 \end{bmatrix} ; ~~D_1=\begin{bmatrix} 0.012 \\ 0.05 \\ 0.036 \\ 0.076 \\ 0.101 \\ 0.332 \end{bmatrix} ; ~~C=\begin{bmatrix} 0&1.2&0&1.75&0&0 \end{bmatrix} \end{aligned}$$

Using the software Scilab, we get the following curves

**Figure 5:**
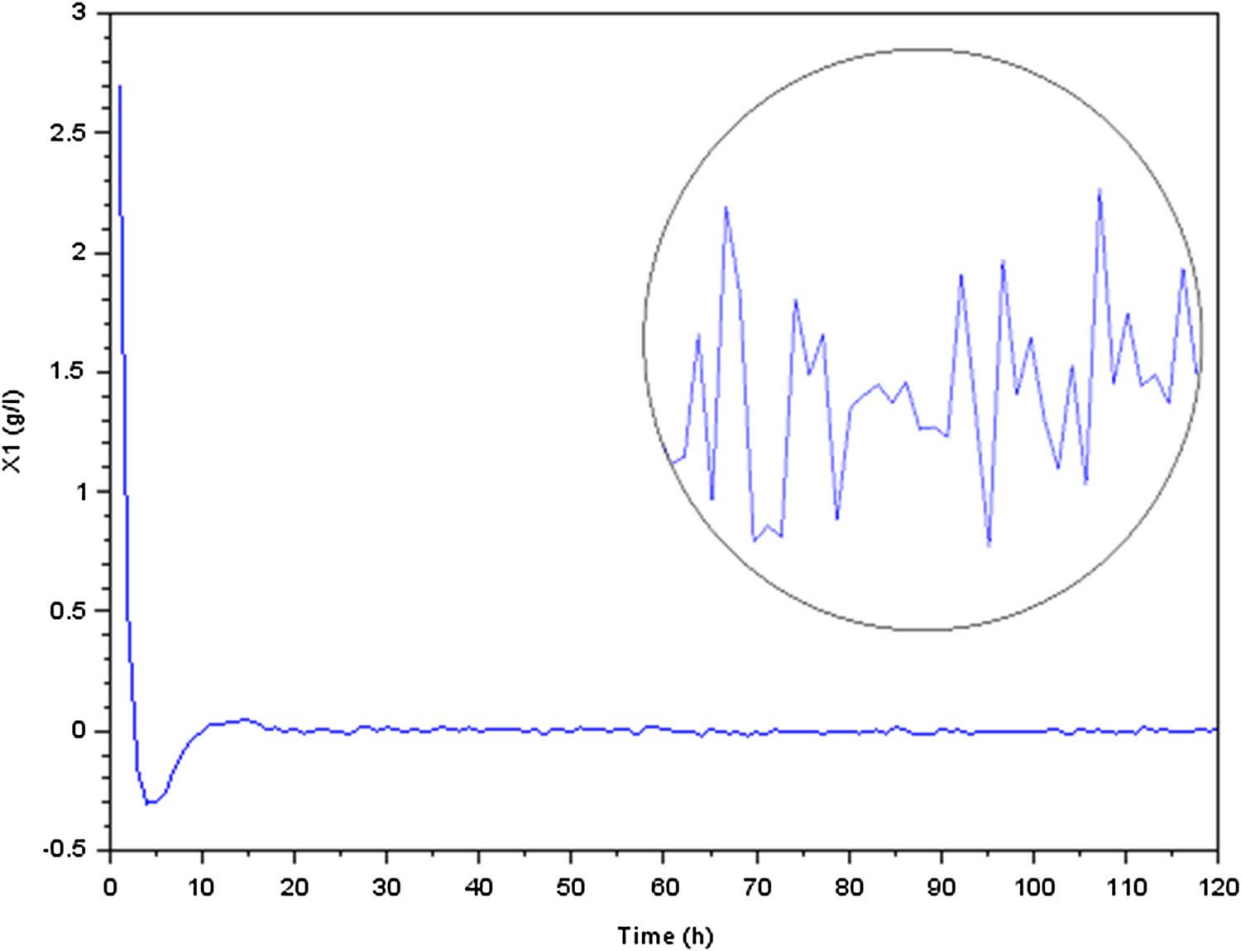
Variation of acidogenic methanogenic bacteria concentration $X_1$ (g/l).

**Figure 6:**
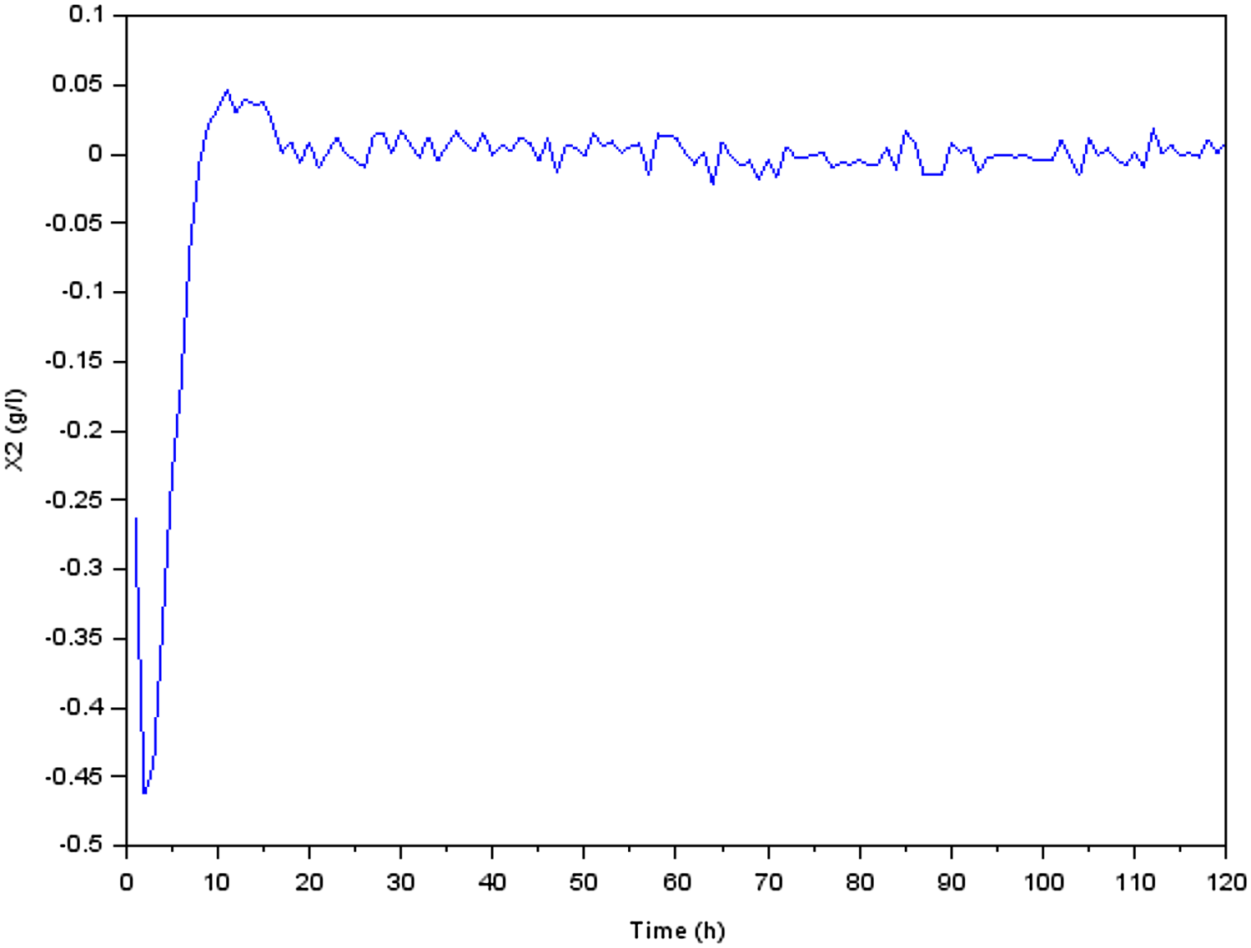
Variation of acetoclastic methanogenic bacteria concentration $X_2$ (g/l).

**Figure 7:**
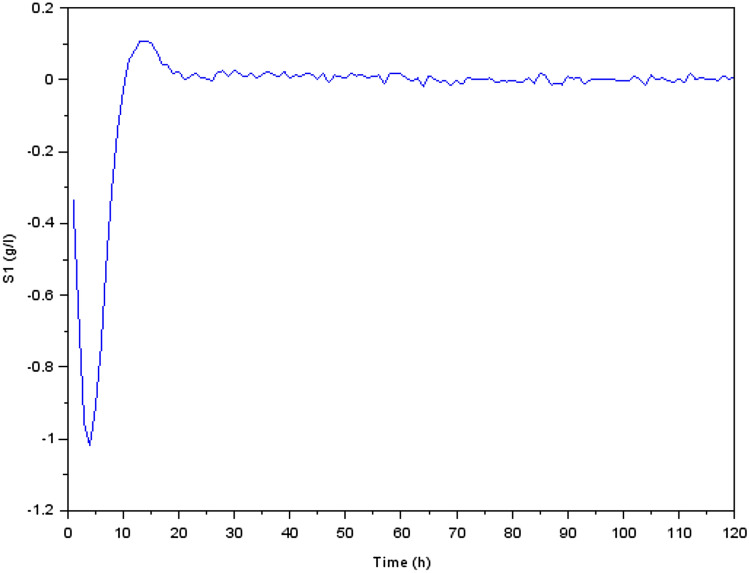
Variation of glucose concentration $S_1$ (g/l).

**Figure 8:**
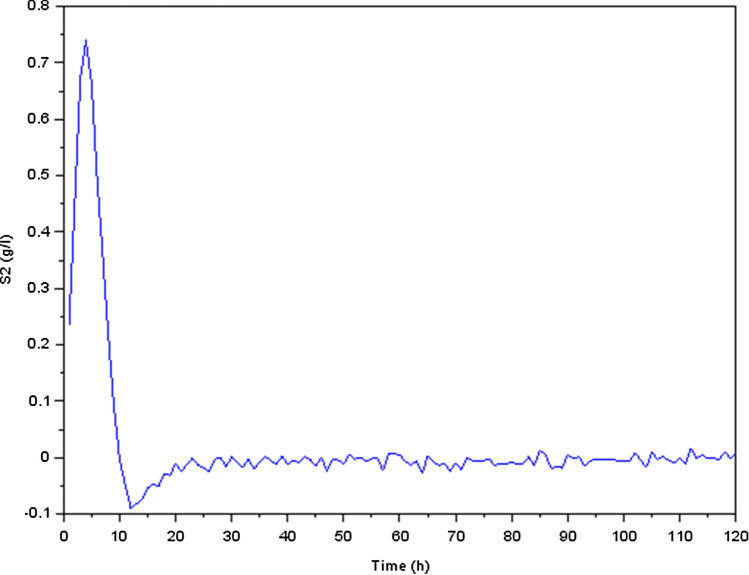
Variation of acetate concentration $S_2$ (g/l).

**Figure 9:**
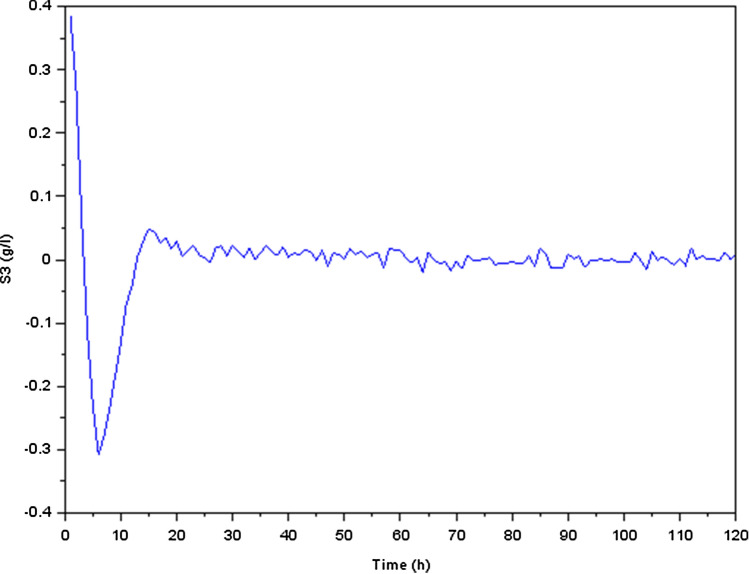
Variation of carbon dioxide concentration $S_3$ (g/l).

**Figure 10:**
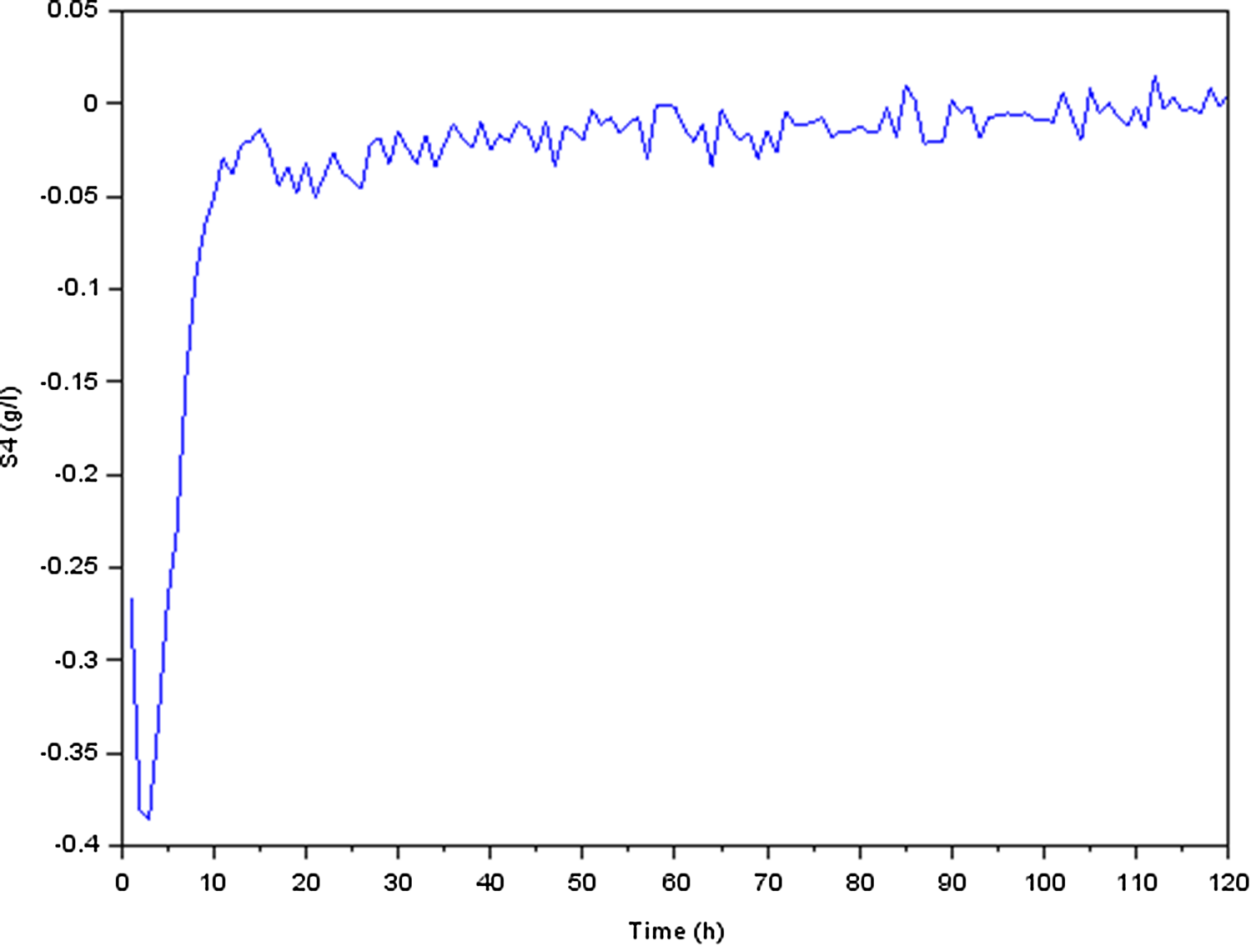
Variation of methane concentration $S_4$ (g/l).

We focused our investigation on the dynamics of a biological process that involves the conversion of glucose to methane during the production process. Our findings were presented as transient reactions of several states of the physical quantities (the state variables). In fact, if the transient response ends up converging towards zero, only the permanent response constitutes the steady state. It’s important to make the transient response converge toward zero and that this response is correctly damped, which is why we have preferred to present the transient responses in Figs. 5, 6, 7, 8, 9 and 10.

We noticed, as shown in Fig. 5 a considerable rise in the concentration of bacterial acidogenic (state $X_1$) during the first phase of the process while ingesting the glucose (state $S_1$), as shown in Fig. 7. Figure 8 demonstrates an increase in $X_1$ was accompanied by the formation of acetate (state $S_2$) and a decrease in carbon dioxide $CO_2$ (state $S_3$), as Fig. 9 indicates. These findings show that the bacteria were metabolizing the glucose to create acetate and other byproducts, resulting in a change in the system’s state.

After around five hours, we observed a loss of controllability, which indicated that the system was no longer stable and was undergoing changes that could not be predicted. This loss of controllability was marked by the disappearance of bacterial acidogenic (state $X_1$) and the appearance of methanogenic bacteria (state $X_2$), as shown in Fig. 6. This shift in the bacterial population indicated that the process was moving towards a different stable state.

This new stable condition, however, was only transitory, as we observed a second loss of controllability after 20 hours, as shown by the absence of methanogenic bacteria (state $X_2$). This discovery revealed that the system was undergoing more unpredictable changes and was transitioning towards a new stable state.

We additionally observed that after around 120 hours, the concentration of methane $CH_4$ (state $S_4$) began to rise, suggesting that the system had entered a new stable state. Overall, our findings indicate that the biological process we investigated was complex, with several transitions between distinct stable states. The transient responses of the state variables gave useful insights into the process dynamics and may be used to improve process performance.

## Conclusion

The purpose of this research was to provide a framework for controlling non-regular singular systems that could not be stabilized using conventional approaches. Using R-Controllability and Imp-Controllability approaches based on Lyapunov’s theory, we investigate admissibility and stability. The synthesis approach adopted for the stabilizing controller is based on disturbance rejection and the consideration of modeling uncertainties of the bounded type in standard that used with robust controls of the $H_2/H_\infty$ type. The algorithms and algebraic transformations are well detailed and lead to the resolution of nonlinear matrix inequalities, which are easy to linearize. The various stages and the intermediate results are confronted at each level with the practical case of operation of the methanizer. The simulations carried out as well as the physical calculations led to a robust stabilization by guaranteeing the asymptotic convergence of the state variation towards zero. Since the transient states converge to zero and the variations are damped, the transition of the system from a stable state to a new stable state is successful by the controller proposed in this paper. In addition, the curves show a perfect description of the physical evolution of the system with the genesis of acidogenic bacteria which, by consuming glucose, acetate and carbon dioxide will eventually give rise to methanogenic bacteria which, in turn, produce methane.

We want to use our findings in a PLC to calculate discrete and hybrid instructions for real-time monitoring of the methane production facility. Our strategy will employ a powerful predictive-type command that also considers potential alerts. Overall, our technique provides a helpful foundation for regulating non-regular singular systems and may be extended to other complex systems.

## Data Availability

The datasets used and/or analysed during the current study available from the corresponding author on reasonable request.
